# HER2 Heterogeneity in Gastric Cancer: A Comparative Study, Using Two Commercial Antibodies

**DOI:** 10.1155/2020/8860174

**Published:** 2020-10-20

**Authors:** Catalin Bogdan Satala, Ioan Jung, Raluca Ioana Stefan-van Staden, Zsolt Kovacs, Calin Molnar, Tivadar Bara, Zsolt Zoltan Fulop, Simona Gurzu

**Affiliations:** ^1^Department of Pathology, George Emil Palade University of Medicine, Pharmacy, Sciences and Technology, Targu-Mures, Romania; ^2^Department of Pathology, Clinical County Emergency Hospital, Targu-Mures, Romania; ^3^Laboratory of Electrochemistry and PATLAB, National Institute of Research for Electrochemistry and Condensed Matter, Bucharest, Romania; ^4^Department of Pathology, George Emil Palade University of Medicine, Pharmacy, Sciences and Technology, Tirgu-Mures, Romania; ^5^Department of Surgery, George Emil Palade University of Medicine, Pharmacy, Sciences and Technology, Tirgu-Mures, Romania; ^6^Department of Surgery, Clinical County Emergency Hospital, Targu-Mures, Romania; ^7^Department of Pathology, Research Center (CCAMF), Tirgu-Mures, Romania

## Abstract

**Background:**

Although amplification of the gene encoding human epidermal growth factor receptor 2 (HER2) is used as an indicator for response to trastuzumab, the reported response rate is low, and few patients with gastric cancer (GC) benefit from this individualized therapy. The aim of this study was to examine the expression of c-erbB-2 oncoprotein (HER2), in GC samples, using two commercial immunohistochemical (IHC) antibodies, and to validate the results by checking *HER*2 gene amplification by fluorescence in situ hybridization (FISH).

**Methods:**

We assessed the IHC expression of HER2 using the polyclonal antibody from Dako and CB11 clone from Leica, in 93 consecutive cases of GC samples. In all of the cases, FISH analysis was also performed using the BOND-MAX platform.

**Results:**

No significant difference was observed between the two HER2 antibodies. Of the 93 cases, 22.58% demonstrated at least focal and 1+ HER2 positivity. Seven cases (7.53%) exhibited 3+ expression, and another 7 carcinomas (7.53%) were equivocal (2+). *HER*2 amplification was seen in 11 cases (11.83%), 10 of which were differentiated adenocarcinomas. In 5 of the cases, 2–5 sections were examined, which proved the extremely high intratumorally/intraglandular heterogeneity. FISH heterogeneity was higher in cases with only 2+ positivity on IHC assessment, compared with those showing at least one small focus of 3+ overexpression. *HER*2 amplification proved to be an independent negative prognostic factor.

**Conclusions:**

Due to the highly heterogeneous aspect of GC, at least 3-4 slides should be assessed by IHC, before considering a tumor to be HER2-negative. In cases with small 3+ foci representing less than 5% of tumor and in equivocal (2+) cases, FISH analysis remains the gold standard method.

## 1. Introduction

Gastric cancer (GC) remains one of the most common causes of cancer-related deaths worldwide, for which the estimation of survival rate, which varies within the same stage, is difficult to be predicted [1]. Although the mortality rate for GC has trended slightly downward in recent decades, it remains a global health problem [2].

Though more than 50 years have passed since the introduction of the Lauren classification, the morphology-based dichotomization of GC into intestinal and diffuse-type carcinoma is still widely used [3]. One of the reasons that an effective targeted therapy has not yet been found for GC is its high heterogeneity not only between patients but also within tumors [4–6]. Intratumorally heterogeneity refers to both morphological aspects and immunoreactivity of tumor cells to antibodies detecting specific biomarkers, such as human epidermal growth factor receptor 2 (HER2) [7].

*HER*2 is a protooncogene located on the long arm of chromosome 17 (17q21), that encodes the transmembrane tyrosine kinase c-erbB-2 oncoprotein, with roles in cellular growth and differentiation. In patients with metastatic GC, HER2 overexpression is used as an indicator of response to anti-HER2 drugs, such as trastuzumab [8–10]. The reasons for the low number of HER2-positive cases and the lack of response to trastuzumab in some positive cases are still unknown. Many commercial antibodies are used for HER2 immunohistochemistry (IHC), and no guideline indicating the number of slides or tumor cells that should be quantified by IHC has yet been implemented.

In this study, we performed an IHC examination of consecutive cases of GC using two anti-HER2 monoclonal antibodies. In cases with inconclusive results, multiple sections were examined, and *HER*2 gene amplification was assessed.

## 2. Materials and Methods

### 2.1. Case Selection

Ninety-three consecutive GC cases diagnosed between 2017 and 2020 in the Department of Pathology of the Clinical County Emergency Hospital, Targu Mures, Romania, were included in the present study. Criteria of inclusion are as follows: patients who received a curative resection, without preoperative adjuvant therapy, with a diagnosis of gastric adenocarcinoma (G1-G3) and a postoperative survival rate of ≥3 months. Poorly cohesive carcinomas, other histological subtypes of carcinomas, nonepithelial or metastatic tumors, and cases from patients receiving palliative surgery were not included. Processing of the cases was approved by the Ethical Committee of the Clinical County Emergency Hospital, Targu Mures, Romania. Written informed consent for publication of clinicopathological data was obtained from patients, who were prospectively included. The follow-up period was between 8 and 42 months.

For all cases, the available slides with tumor cells were reanalyzed. We aimed to establish the staging according to the most recent edition of the American Joint Committee on Cancer tumor staging manual [11]. Tumors were also staged according to the Dukes-MAC-like staging system, proposed in 2017 [12].

### 2.2. Immunohistochemistry Analysis and Interpretation

In all cases, conventional slides were used for IHC assessment. After reviewing of the hematoxylin and eosin-stained sections, two experienced pathologists chose one representative sample to be used for further IHC processing. For all 93 cases, we performed immunostaining for HER2 using two monoclonal antibodies from two different manufacturers: Dako (DakoCytomation, Glostrup, Denmark) and Leica (Leica Biosystems, Germany). We chose the polyclonal antibody c-ErbB-2 (HER2) from Dako and CB11 clone from Leica. High pH retrieval was performed for the two antibodies. The concentrated antibody from DAKO was diluted (1 : 800), but the Leica antibody was ready to use (RTU). Immunostaining was performed automatically (for both antibodies) using the Bond Max fully automated IHC stainer (Leica).

After developing with diaminobenzidine (DAB) and counterstaining with hematoxylin, the membrane expression of HER2 was independently evaluated by two experienced pathologists based on the HercepTest^TM^ guideline and Ruschoff's criteria [13]: score 0 (negative), tumor cells showed no reactivity or showed reactivity in a site other than the membrane; score 1 (negative), barely visible complete, basolateral, or lateral membranous reaction, visible only at 40x magnification, in ≥10% of cells; score 2 (equivocal), weak to moderate complete, basolateral, or lateral membranous reaction visible at 10–20x magnification, in ≥10% of tumor cells; score 3 (positive), strong complete, basolateral, or lateral membranous staining, in ≥10% of tumor cells. In cases in which the results differed between the two pathologists, the case was reevaluated by both pathologists and by the senior pathologist on the team. When necessary, immunostaining was performed on supplementary slides, for elucidation. For cases showing heterogeneous immunostaining (e.g., small areas with 3+ positivity, below 5–10%, surrounded by areas with 2+ positivity), the percentage of each grade was determined, and *HER*2 gene amplification was assessed by fluorescence in situ hybridization (FISH).

### 2.3. FISH Analysis and Interpretation

To evaluate the grade of *HER*2 gene amplification and establish an in-house protocol, all HER2-positive tumors, independent of the IHC grade (1+, 2+, and 3+), were further assessed by FISH. FISH analysis was also performed in samples that showed positivity with only one of the two antibodies.

The FISH technique was automatically performed using the Bond Max fully automated IHC and FISH stainer (Leica). It was performed using the PathVysion HER2 DNA Probe Kit according to the manufacturer's instructions. For interpretation, we used the LSI HER2/neu spectrum orange/chromosome 17 centromere probe (CEP17)/spectrum green on a Leica CytoVision system based on a Leica DM4000 fluorescence microscope. The analysis considered 30–50 cells from the hotspot, which were chosen at low magnification and then counted at x1000 magnification. Cases with a HER2/CEP17 ratio under 1.8 were considered negative and those with a ratio ≥2.2 were classified as positive. In cases with a HER2/CEP17 ratio between 1.81 and 2.19 and in negative cases, the count was performed again, first by the same pathologist and then by a pathologist experienced in FISH interpretation, in collaboration with a molecular geneticist, with further correlation of results. In addition, in cases that were either negative or equivocal, 50–100 cells were examined for the second interpretation. When necessary, FISH analysis was performed on supplementary slides, for elucidation.

### 2.4. Statistical Analysis

The results were further analyzed using GraphPad Prism 8 (software-free version). The correlation between the overall survival rate, the clinicopathological parameters, and the grade of IHC staining for HER2 was performed using Fisher's exact test and the chi-square test. For all analyses, *p* values less than 0.05 were considered statistically significant (95% confidence interval).

## 3. Results

### 3.1. Clinicopathological Parameters

The 93 patients included in the present study were diagnosed with GC between the ages of 47 and 83, and the male-to-female ratio was 2.44. Most of the cases were G2 (moderately differentiated) or G3 (poorly differentiated) adenocarcinomas. Half of the cases (*n* = 55; 59.13%) were diagnosed in the advanced stage, pT4N0-3 (Dukes-MAC-like stage D). The other cases were staged as follows: 21.50% (*n* = 20) as C2 (T3N1-3), 4.30 (*n* = 4) as C1 (T3N0), none as B2 (T2N1-3), 6.45% (*n* = 6) as B1 (T2N0), 1.07% (*n* = 1) as A2, and 7.52% (*n* = 7) as A1 (Table 1).

### 3.2. Immunohistochemical Assessment of HER2

Of the 93 tumors analyzed, 22.58% (*n* = 21) demonstrated focal positivity of at least 1+, independent of the antibody used. Only 7.53% of cases (*n* = 7) was assessed as 3+ (positive) using the Dako antibody, and 6.45% (*n* = 6) was assessed as 3+ using the Leica antibody. In 7.53% of cases (*n* = 7), the IHC assessment showed 2+ positivity (equivocal) using both the Dako and the Leica antibodies. The 2+ category contained the same number of tumors for both clones due to the underscoring tendency of the Leica compared to the Dako antibody: one 3+ case according to Dako assessment was underscored as 2+ using the Leica antibody, and one 2+ case according to Dako was underscored as 1+ using the Leica antibody. In the category of 1+ (negative), we identified 7.53% of cases (*n* = 7) using the Dako and 8.60% (*n* = 8) using the Leica antibodies (Table 2).

### 3.3. Fluorescence In Situ Hybridization Assessment of HER2

FISH analysis demonstrated *HER*2 gene amplification in all cases assessed as 3+ on IHC, while all cases reported as 1+ (negative) on IHC were confirmed to lack *HER*2 amplification.

**Figure 1:**
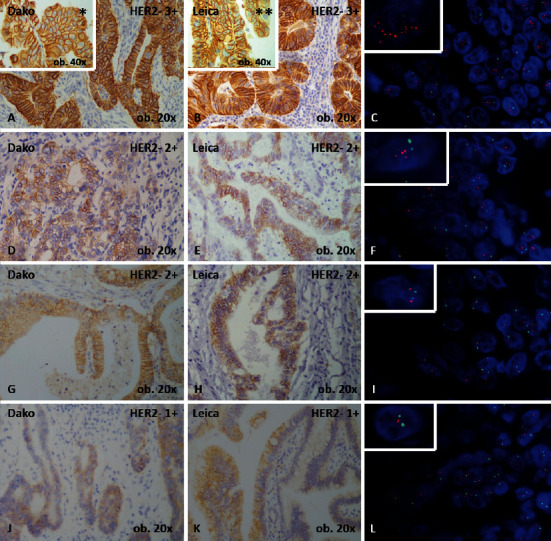
Immunoexpression of c-erbB-2 oncoprotein (HER2), revealed with two commercial antibodies and the corresponding FISH examination. In cases with HER2 3+ positivity (A and B), with strong, evident, complete membranous staining, and well visible on both 20x and 40x magnification (∗ and ∗∗), the *HER*2 gene amplification is clearly proved by FISH analysis (C). In some cases with HER2 2+ positivity (D and E), with moderate complete, membranous reaction visible at 20x magnification, *HER*2 amplification is present (F), whereas other HER2 2+ cases (G and H) do not show amplification (I), same as the cases assessed as 1+ (J-L).

The case reported as 3+ on IHC using the Dako antibody and 2+ using the Leica antibody also showed *HER*2 gene amplification. Of the 7 cases with equivocal (2+) results using the Dako clone, 4 showed *HER*2 gene amplification (Figure 1).

While the IHC assessment confirmed HER2 positivity (3+) in 7.53% (*n* = 7) and 6.45% (*n* = 6) of cases using the Dako and Leica antibodies, respectively, and 2+ positivity (equivocal) in another 7.53% of cases (*n* = 7), *HER*2 gene amplification was demonstrated in 11.82% of cases (*n* = 11). The results are summarized in Table 2.

### 3.4. Intratumorally Heterogeneity

To assess tumor heterogeneity, for five of the cases, we evaluated HER2 expression by IHC and *HER*2 gene status by FISH on all available slides with viable tumor tissue without extensive necrosis or hemorrhage.

The first two cases were G2 adenocarcinomas with no known distant metastases. Here, 3+ HER2 positivity was found in over 50% of tumor cells on all four slides examined for each case with both of the antibodies. *HER*2 gene amplification was confirmed by FISH analysis.

**Figure 2:**
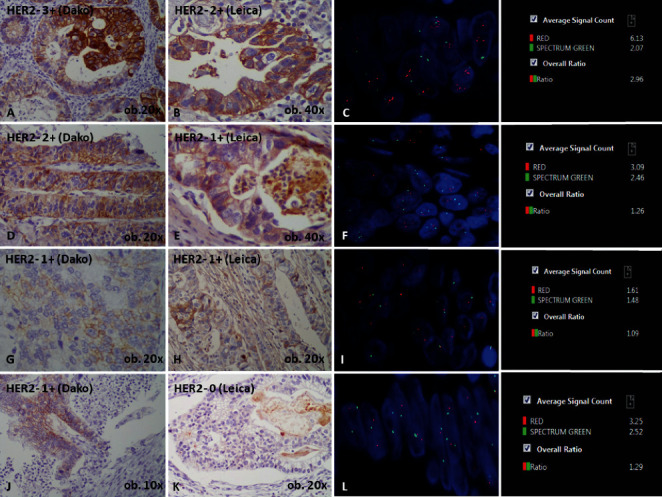
Multiple sections from a representative metastatic gastric adenocarcinoma, showing intratumor/intraglandular heterogeneity for HER2 expression and its corresponding FISH expression. One of the slides from primary tumor shows a 3+ focus with Dako (A) which was equivocal (2+) with Leica stain (B) and confirmed as amplified (C) with a HER2/CEP17 ratio of 2.96. In a second slide (D and E), no gene amplification is seen (F), same as for the third section from primary tumor (G–I). The hepatic metastatic tissue also shows no HER2 positivity (J, K) and no gene amplification (L), independently from the used antibody.

The third case was a G2 adenocarcinoma with hepatic metastases, from which 4 sections from the primary tumor and one from hepatic metastatic tissue were processed. On IHC assessment of this particular case, the first of four tumor sections from the primary tumor demonstrated 3+ HER2 expression on a single focus, representing less than 5% of the tumor cells, using the antibody from Dako, with the same spot expressing HER2 at a grade of 2+ using Leica assessment. Of the remaining three tumor sections, one demonstrated 2+ HER2 expression using the Dako clone, while the corresponding analysis with the Leica antibody showed only 1+ expression. The other two sections from the primary tumor were graded as 1+ using both the Dako and Leica antibodies, and the one section derived from metastatic tumor tissue was negative. FISH analysis demonstrated *HER*2 amplification only on the first slide that demonstrated positive/equivocal HER2 expression by IHC, with no amplification observed for the remaining slides, including the slide with metastatic tumor tissue (Figure 2).

**Figure 3:**
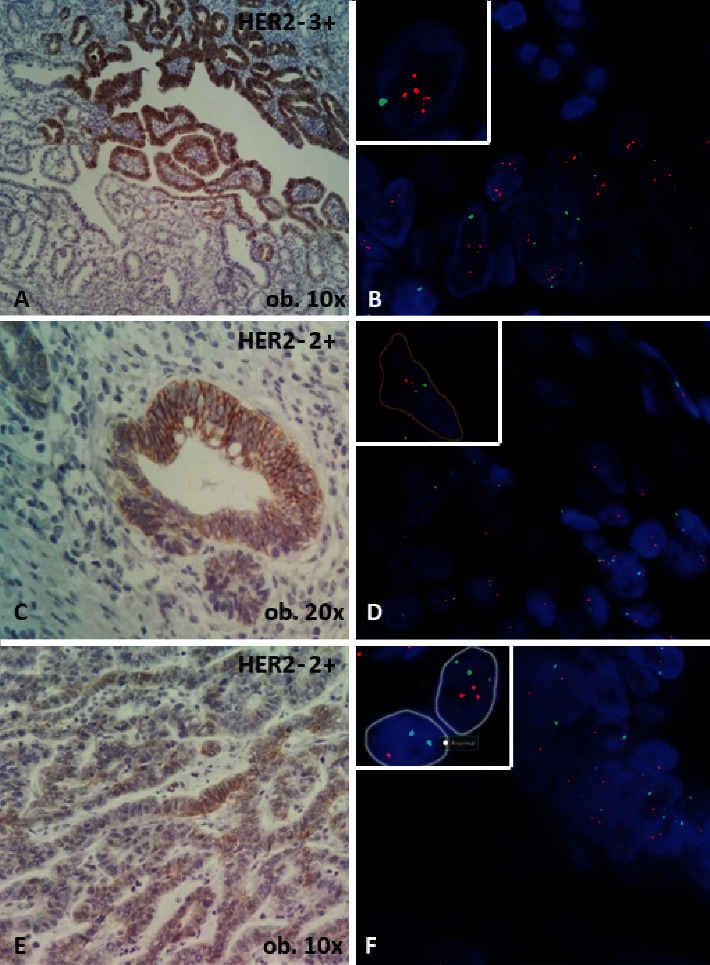
Intratumor heterogeneity, revealed by both immunohistochemical and FISH assessment. In one slide, tumor cells exhibit one focus of 3+ expression (A), confirmed as *HER*2*-*amplified, with a HER2/CEP17 ratio of 2.41 (B). In other two sections (C–F), extensive areas of 2+ expression can be seen (C and E), with no *HER*2 gene amplification (D and F).

The fourth case was a G2 adenocarcinoma with no known distant metastases. HER2 assessment by IHC exhibited obviously heterogeneity, with one section showing a focus of 3+ expression, which represent below 5% of tumor cells, proved amplified on FISH analysis. The rest of two assessed sections demonstrated equivocal expression on IHC (2+), and they were certified as nonamplified on FISH assessment (Figure 3).

**Figure 4:**
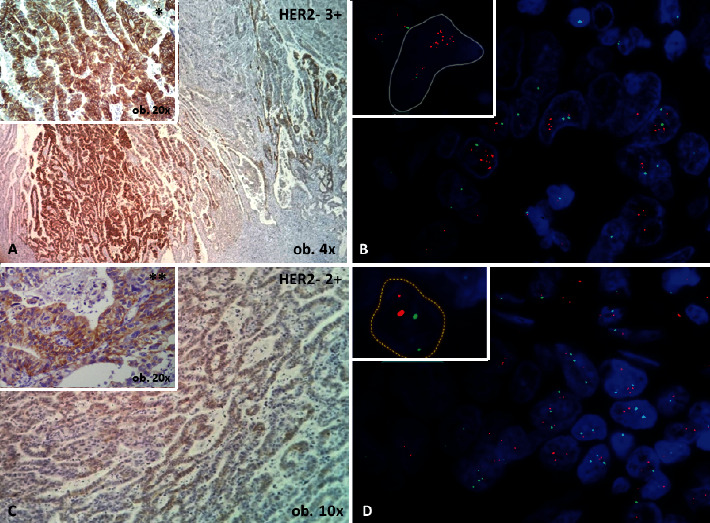
Two sections from a gastric adenocarcinoma, with HER2 heterogeneity. On the sample with a single focus of 3+ expression on immunohistochemistry (a), the *HER*2 gene is amplified, with a HER2/CEP17 ratio of 2.21 (b). On the second sample, with larger areas of 2+ positivity (c), no gene amplification is proved on FISH (d).

Last, but not least, the fifth case was a G2 adenocarcinoma with multiple regional lymph node metastases (pN3), but no known distant metastases. IHC assessment demonstrated a heterogeneous pattern, with one slide with foci of 3+ expression, below 5%, which were confirmed as *HER*2*-*amplified. The second slide showed that multiple areas of 2+ positivity (over 30%) were proved as nonamplified on FISH analysis (Figure 4).

### 3.5. Correlation of HER2 Expression with Clinicopathological Parameters

**Figure 5:**
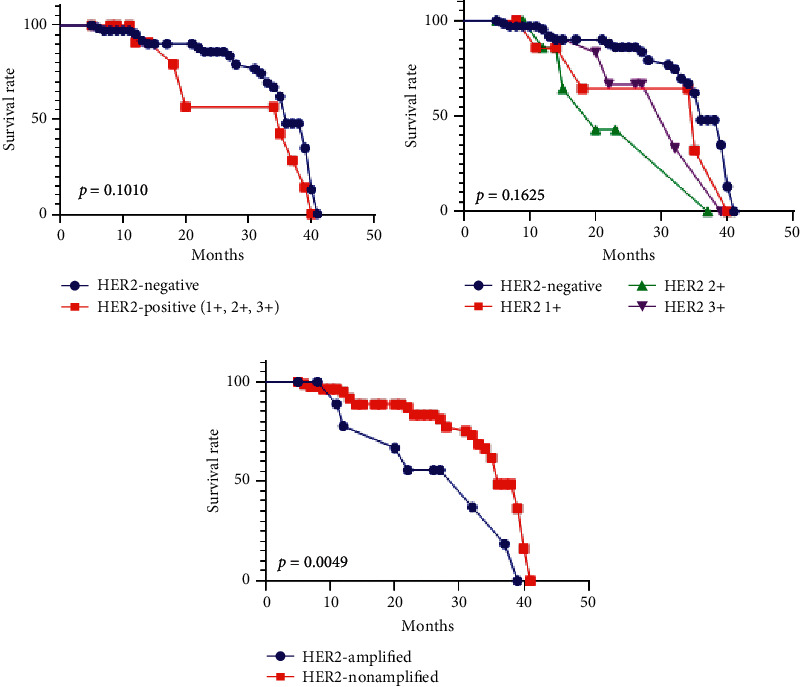
Kaplan–Meier survival curves demonstrate no independent prognostic role of the HER2 immunoexpression (a) and (b), but cases confirmed by FISH with *HER*2 gene amplification have a considerably lower survival rate, compared to the nonamplified cases (c).

Examination of the demographic parameters and tumor-related parameters (such as localization, depth of tumor infiltration, lymph node status, lymphovascular invasion, or presence of distant metastases) did not exhibit correlation with the rate of *HER*2 amplification. Most of the amplified cases (10/11) were differentiated adenocarcinomas (G1/2), with only one of the 50 G3 adenocarcinomas exhibiting amplified *HER*2 (Table 3). The overall survival rate was also not correlated with the expression of HER2 by IHC. In contrast, FISH-verified amplification of the *HER*2 gene was an independent indicator of worse survival (Figure 5).

## 4. Discussion

Despite improvements in the diagnosis and treatment of patients with GC, the 5-year survival rate is still poor, only 30%–35% [9, 10]. With many GCs diagnosed every year, the need for standardized prognostic and predictive markers is emphasized in many studies published on this subject; nonetheless, much remains unknown [14, 15]. Amongst the markers studied in GC, HER2 seems to have the greatest importance not only as a prognostic marker but also because it has therapeutic importance due to the development and use of anti-HER2 therapy [16, 17]. Trastuzumab is the only anti-HER2 target therapy approved in GC [17], but the selection of patients that could benefit from this treatment is not as straightforward as it is in breast cancer.

The main reason of the difficulty in assessing HER2 in GCs is the intratumorally heterogeneity of its expression, which occurs in 69%–75% of cases [4–7, 9, 18, 19]. In this paper, we emphasize and confirm this heterogeneity, which is present in the same tumor, between primary tumor and metastatic tissue and even in the same tumor gland. It is difficult to manage this aspect, as usually only one tumor section is used for diagnosis, and the cutoff is 10%. This paper highlights the importance of testing HER2 expression in at least 3-4 slides, especially for differentiated carcinomas that do not show 3+ positivity on the first slide. Moreover, if tumor cells express 3+ or 2+ HER2 at any extent, even under 5%, on the first slide, it is worth analyzing additional tumor slides for larger foci of HER2 positivity. No cases should be considered HER2-negative without IHC examination of at least 3-4 slides. Biopsy specimens should not be interpreted as negative in any cases, and at least 5 different fragments should be analyzed [9, 20].

In this study, 15% of cases were HER2-positive (2+ and 3+), in line with literature data that showed relatively wide ranges of HER2 protein expression (between 5% and 42%) [21]. The amplification rate was 11.83% in this cohort, the reported rate being from 4% to 13% [21]. These relatively wide ranges are due to the different protocols using, beginning with discrepancies in fixation, use of different antibodies and, maybe most important, use of nonstandardized scoring protocols, especially on FISH analysis. Like our data, it was previously emphasized that the percentage of HER2 overexpression is consistently higher in tumors with well- or moderately differentiated morphology compared to poorly differentiated carcinomas [22–26]. As poorly cohesive carcinomas rarely express HER2, we did not include such cases in this study. However, their inclusion might significantly decrease the reported rate of HER2 positivity.

With the well-known possibility of false-positive/false-negative results on IHC assessment, we simultaneously evaluated the cases under the study using two different commercial HER2 antibodies. The positivity rate was similar, with only one of the 3+ cases using the Dako antibody showing equivocal positivity (2+) with the Leica antibody. However, this case showed *HER*2 amplification. Moreover, all 1+ cases identified using the Dako antibody were assessed as negative using the Leica antibody. As the correlation between the results obtained using the antibodies from the two manufacturers is over 90% [21, 26–29], both clones can be safely used in daily diagnosis, but an in-house standardization is mandatory.

An interesting aspect arose regarding the impact of IHC heterogeneity on FISH analysis, which should be performed by an experienced pathologist. In cases that showed at least one focus of 3+ overexpression on IHC with either of the clones used, with larger areas of 2+ IHC positivity nearby, FISH analysis demonstrated a relatively homogeneous number of amplified *HER*2 copies in both the 3+ and the 2+ areas. In comparison, in FISH-confirmed positive cases with only 2+ positivity on IHC assessment, FISH analysis demonstrated intercellular heterogeneity in the number of amplified *HER*2 copies. From our perspective, this could have two possible explanations: either the IHC assessment was performed with too much vigilance, with 3+ areas being misinterpreted as 2+ areas due to technical difficulties, or the subcellular mechanisms responsible for *HER*2 amplification are slightly different in cases with 3+ IHC results compared to those with only 2+ expression. These aspects should raise the possibility of other molecular signaling pathways acting as positive modulators of classic *HER*2 gene expression. It was even suggested that a better response to trastuzumab could be obtained in patients whose tumors exhibited 3+ HER2 (quantified using IHC methods and confirmed with *HER*2 amplification) compared with equivocal (2+) amplified cases [30].

One molecular mechanism involved in GC cell heterogeneity could be polysomy, whole chromosomal multiplication [31–34], but this subcellular alteration cannot be responsible for molecular modification in all cases, as FISH analysis does not always report equal amplification of the centromeric region of chromosome 17 (CEP17) and the *HER*2 gene.

## 5. Conclusions

In GC, the *HER*2 gene is more frequently amplified in differentiated adenocarcinomas, but the rate of intratumorally heterogeneity is extremely high. To prove HER2 positivity, at least 3-4 slides should be examined, and FISH analysis should be performed in any case that shows clusters of HER2 3+ positivity, even they represent less than 5% of tumor cells. A gastrointestinal pathologist with experience in FISH analysis should perform interpretation of immunostaining. Both Dako and Leica clones can be successfully used in daily practice. When possible, for FISH analysis, the samples with at least one small focus of 3+ should be chosen, rather than those with extensive 2+ positivity. *HER*2 amplification is an independent negative prognostic indicator in GC.

## Figures and Tables

**Table 1:** Clinicopathological parameters of the included cases.

Parameter		Values (*n* = 93)
Median age (years)		67 ± 11.45 (range 4783) (%)

Gender	Males	66 (70.96)
	Females	27 (29.04)

Histologic subtype and grade	Well-differentiated adenocarcinoma (G1)	4 (4.30)
	Moderately differentiated adenocarcinoma (G2)	39 (41.93)
	Poorly differentiated adenocarcinoma (G3)	50 (53.76)

Depth of invasion (pT stage)	pT1	8 (8.60)
	pT2	6 (6.45)
	pT3	24 (25.80)
	pT4	55 (59.13)

Lymph node status (pN stage)	pN0	17 (18.27)
	pN1-3	76 (81.73)

Distant metastases (pM stage)	pM0	76 (81.73)
	pM1	17 (18.27)

Dukes-MAC-like Stage
	*A1*	7 (7.52)
	*A*2	1 (1.07)
	*B1*	6 (6.45)
	*B*2	—
	*C1*	4 (4.30)
	*C*2	20 (21.50)
	*D*	55 (59.13)

**Table 2:** Correlation between immunohistochemical results regarding c-erbB-2 oncoprotein (HER2) expression, using two commercial antibodies and gene status.

Immunostains		Total cases (number/%)	FISH assessment-*HER*2 gene status (number/%)	
			Amplified cases	Nonamplified cases

HER2 3+	Dako polyclonal	7/33.33	7/100%	0
	Leica CB11 clone	6/28.57	6/100	0

HER2 2+	Dako polyclonal	7/33.33	4/57.14	3/42.86
	Leica CB11 clone	7/33.33	5/71.42	2/28.58

HER2 1+	Dako polyclonal	7/33.33	0	7/100
	Leica CB11 clone	8/38.09	0	8/100

**Table 3:** Correlation between clinicopathological factors and HER2 gene status.

Parameter		HER2 gene status		*p* value
		Number of amplified cases	Number of nonamplified cases	

Median age (years)		73 ± 11.13	67 ± 15.32	0.57

Gender	Male	7	59	0.72
	Female	4	23	

Localization	Proximal stomach	5	38	1.00
	Distal stomach	6	44	

Histological grade	G1	3	1	**<0.001**
	G2	7	32	
	G3	1	49	

pT stage	pT1-2	1	13	0.64
	pT3	4	20	
	pT4	6	49	

pN stage	pN0	3	18	0.71
	pN1-3	8	64	

pM stage	pM0	9	67	1.00
	pM1	2	15	

Dukes-MAC-like stage	A1 (T1N0) + B1 (T2N0) + C1 (T3N0)	3	14	0.58
	A2 (T1N1-3) + C2 (T3N1-3)	3	18	
	D (T4N0-3)	5	50	

Lymphovascular invasion	Present	9	51	0.32
	Absent	2	31	

## Data Availability

The clinicopathological data used to support the findings of this study are available from the corresponding author upon request.
